# Green manure (*Ophiopogon japonicus*) cover promotes tea plant growth by regulating soil carbon cycling

**DOI:** 10.3389/fmicb.2024.1439267

**Published:** 2024-09-18

**Authors:** Shuaibo Shao, Zhongwei Li, Yanqi Zhu, Yi Li, Yuanping Li, Linkun Wu, Christopher Rensing, Pumo Cai, Caihao Wang, Jianmin Zhang, Qisong Li

**Affiliations:** ^1^College of Tea and Food, Wuyi University, Wuyishan, China; ^2^College of Resources and Environment, Fujian Agriculture and Forestry University, Fuzhou, China; ^3^Institute of Environmental Microbiology, Fujian Agriculture and Forestry University, Fuzhou, China; ^4^College of Life Sciences, Fujian Agriculture and Forestry University, Fuzhou, China

**Keywords:** green manure, tea plant, soil microbiota, high-throughput sequencing, *Ophiopogon japonicus*

## Abstract

**Introduction:**

In mountainous tea plantations, which are the primary mode of tea cultivation in China, issues such as soil erosion and declining soil fertility are particularly severe. Although green manure cover is an effective agricultural measure for restoring soil fertility, its application in mountainous tea plantations has been relatively understudied.

**Methods:**

This study investigated the effects of continuous green manure cover using the slope-protecting plant *Ophiopogon japonicus* on tea plant growth and soil microbial community structure. We implemented three treatments: 1 year of green manure coverage, 2 years of coverage, and a control, to study their effects on tea plant growth, soil physicochemical properties, and soil bacterial and fungal communities.

**Results:**

Results demonstrate that green manure coverage significantly promote the growth of tea plants, enhanced organic matter and pH levels in soil, and various enzyme activities, including peroxidases and cellulases. Further functional prediction results indicate that green manure coverage markedly promoted several carbon cycling functions in soil microbes, including xylanolysis, cellulolysis, degradation of aromatic compounds, and saprotrophic processes. LEfSe analysis indicated that under green manure cover, the soil tends to enrich more beneficial microbial communities with degradation functions, such as *Sphingomonas*, *Sinomonas*, and *Haliangium* (bacteria), and *Penicillium*, *Apiotrichum*, and *Talaromyce* (fungi). In addition. Random forest and structural equation models indicated that carbon cycling, as a significant differentiating factor, has a significant promoting effect on tea plant growth.

**Discussion:**

In the management practices of mountainous tea plantations, further utilizing slope-protecting plants as green manure can significantly influence the soil microbial community structure and function, enriching microbes involved in the degradation of organic matter and aromatic compounds, thereby positively impacting tea tree growth and soil nutrient levels.

## 1 Introduction

Tea plant (*Camellia sinensis.* L.), a globally popular beverage, is extensively cultivated across China (Yuan et al., 2018). Mountainous tea plantations are one of the main models of tea plantations in China, with over 180,000 hm^2^ in Fujian Province alone (Wu et al., 2023). However, significant issues such as soil erosion and soil fertility decline due to terraced planting remain prominent. In recent years, soil fertility degradation of moderate and above levels in Fujian’s mountainous tea plantations has exceeded 63,000 hm^2^, accounting for 33.42% of the total tea garden area (Liu et al., 2019), leading to a noticeable decline in tea yield and quality (Shen and Lin, 2021; Wu et al., 2021). Therefore, there is an urgent need for new agricultural strategies to mitigate the adverse impacts on mountainous tea plantations. Green manure incorporation is a critical component of sustainable agricultural development, playing an irreplaceable role in improving soil environments, reducing the risk of soil erosion, and controlling weeds (Campanella et al., 2020; Liu Q. et al., 2021). Previous studies have shown that green manure can reduce soil moisture evaporation, thus reserving water for subsequent crop seasons and significantly enhancing the efficiency of nitrogen fertilizer application (Gabriel et al., 2016). Additionally, green manure contributes to the accumulation of soil organic matter, enhancing soil carbon sequestration and nutrient mineralization capabilities (Wang et al., 2016; Zhang et al., 2019). Increasingly, research suggests that these advantageous mechanisms result from interactions among plants, soil, and microbes, where changes in the soil microbial community structure and function mediated by green manure are key factors in realizing these benefits (Li et al., 2020; Wang et al., 2022).

Soil organic matter represents the largest store of organic carbon on Earth and is a major source of nutrients (Crowther et al., 2019). Soil microbes play a critical role in shaping the cycling of organic matter, thereby impacting ecosystem functions such as carbon sequestration, nitrogen fixation, and stress resistance, significantly affecting the surrounding environment and climate (Liang et al., 2019; Angst et al., 2021). Green manure incorporation primarily enhances the soil environment through interactions between soil nutrients and microbial community (Balachandar et al., 2020). For instance, the incorporation of alfalfa not only increases the diversity of soil microbes but also significantly enhances the abundance of Proteobacteria, promoting plant growth (Ai et al., 2020). Similarly, the use of barley and hairy vetch as cover crops enriches the population of actinobacteria in rice soils, leading to increased rice yields (Khan et al., 2020). The increase in soil microbial diversity induced by green manure can enhance soil enzyme activity and nutrient content (Chavarría et al., 2016). The reason is that when the roots and above-ground parts of green manure plants are turned into the soil and decompose, they form humus, which provides a more diverse carbon source input for soil microorganisms, inevitably having a profound impact on the ecological functions of the soil microbial community (Francesca Cotrufo et al., 2021). Therefore, changes in crop yield and quality under green manure treatment are closely related to changes in the structure of the soil microbial community.

In the context of tea cultivation, the incorporation of legume crop residues has been shown to adjust the balance of exchangeable cations, thereby alleviating soil acidification in tea plantations (Wang et al., 2009). Furthermore, numerous studies have demonstrated that the combined application of animal manure and crop residues significantly enhances soil nutrient content and promotes the cycling of carbon and nitrogen in tea plantation soils (Tayyab et al., 2018; Jiang et al., 2019). Thus, it is evident that green manure plays a role in improving the soil nutrient environment for tea plants. However, in the terrain of mountainous tea plantations, these leguminous plants face challenges such as difficulty in management and high costs, making their practical application in mountainous tea plantations relatively limited (Tao et al., 2021). Therefore, it is particularly important to explore a green manure cover model that is more compatible with the terraced terrain of mountainous tea plantations and similar to the growth habits of tea plants.

*Ophiopogon japonicus*, a monocotyledonous perennial herbaceous medicinal plant, shares similar growing conditions with tea plants and possesses several advantages such as pest resistance, drought tolerance, and cold hardiness (Chen et al., 2021). Utilizing *O. japonicus* as a slope-protecting plant in tea plantations can effectively prevent soil erosion (Li et al., 2021). Most importantly, *O. japonicus* can easily grow on the terrace walls of mountainous tea plantations, forming a natural high-low ecological niche advantage. Further research on its application as green manure in tea plantations is expected to reduce farmers’ fertilizer input in the spring and autumn seasons, enhance soil ecological stability, and increase tea yield. This has important theoretical and practical significance for the sustainable development of tea plantations. Therefore, we conducted field experiments in the National Soil and Water Conservation Demonstration Plantation in Wuyishan City, Fujian Province, to explore the effects of continuous use of *O. japonicus* as green manure on soil nutrients, microbial community structure, and function in tea plantations. This study aims to provide a theoretical foundation and technical guidance for the promotion of green manure cover management practices in mountain tea plantations.

## 2 Materials and methods

### 2.1 Overview of the research area

**Figure 1:**
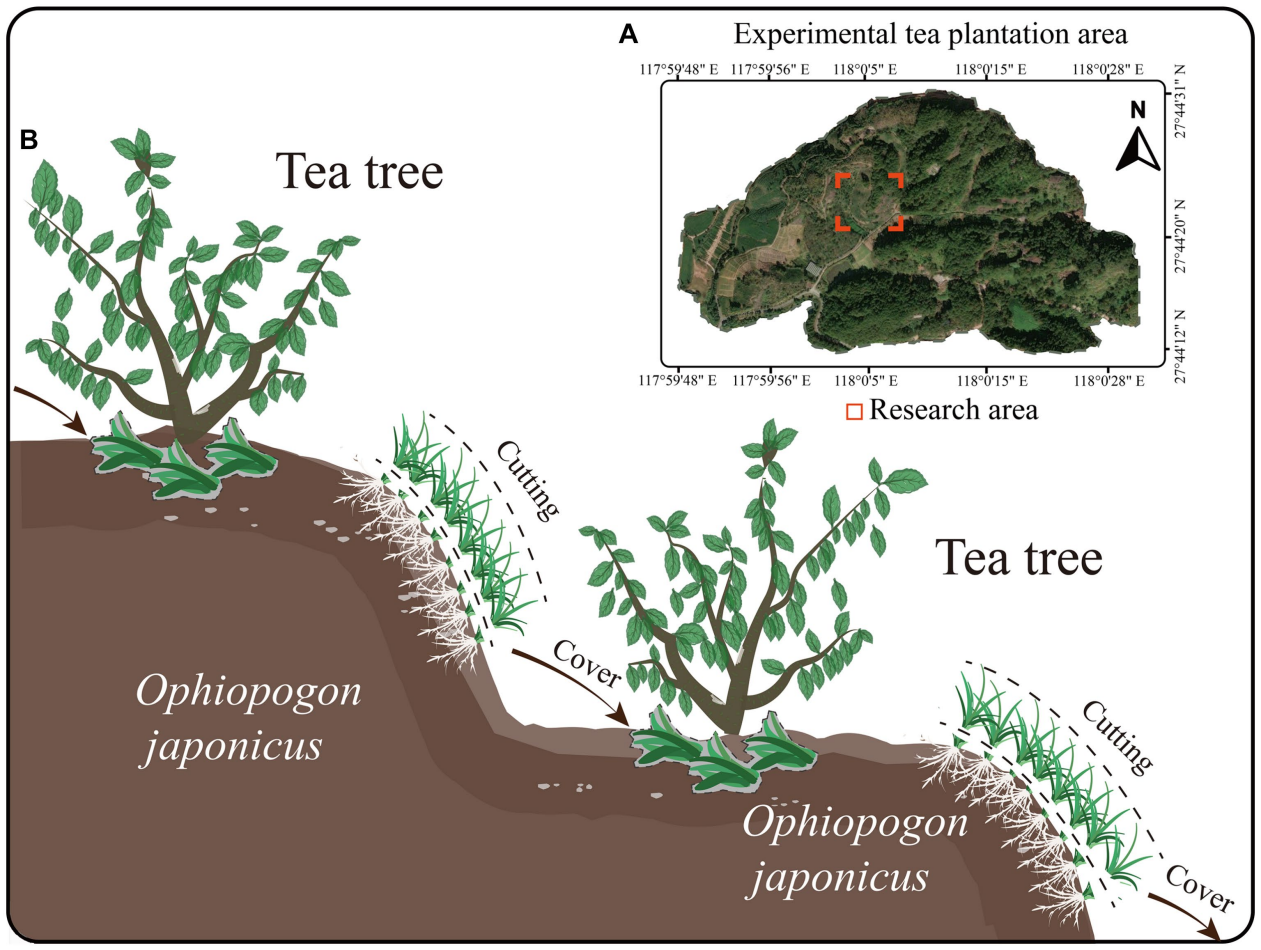
Research area and green manure cover diagram. **(A)** Research area. **(B)** Green manure cover diagram.

The experimental site is located at the National Soil and Water Conservation Demonstration Plantation of Wuyi University in Wuyishan City, Fujian Province, China (longitude 118°00′5″E, latitude 27°44′25″N) (Figure 1A). The tea plantation is arranged in terraces (with a slope of 18°) and is situated in a subtropical monsoon humid climate zone, with an annual average rainfall of 1926.9 mm. The experimental tea plantation covers an area of 13.2 ha, and the tea variety used is the five-year-old Rougui (*Camellia sinensis.* L. Rougui). The green manure applied in the study is *Ophiopogon japonicus*.

### 2.2 Experimental design

The intercropping experiment with tea trees and *O. japonicus* began in April 2018, with the *O. japonicus* planted on the terraces and terrace walls of a mountain tea plantation. A vegetation cover survey of the tea plantation found that, 2 years into the intercropping (2020), the coverage of *O. japonicus* reached high levels (over 68% in April, 83% in August, and 81% in December, Supplementary Figure S1). Therefore, starting from April 2021, an experiment using *O. japonicus* as green manure was conducted. The experiment was set up with three treatments: intercropping without cutting the *O. japonicus* (CK), cutting the *O. japonicus* after 1 year of coverage (OC1), and cutting after 2 years of coverage (OC2). Each treatment had three plots measuring 20 m × 5 m. From 2021 to 2022, the *O. japonicus* was cut in April, August, and December each year, with a residual height of 3 cm. The cut *O. japonicus* was evenly distributed over the roots of the tea plants, with an average coverage of 0.825 kg/m^2^ (dry weight 0.372 kg/m^2^) (Figure 1B). The baseline soil fertility (0–20 cm) was measured as follows: pH 4.35, total nitrogen 0.64 g/kg, total phosphorus 0.58 g/kg, total potassium 9.12 g/kg, and organic matter 12.56 g/kg. Cultural management practices in the different tea gardens were kept consistent, with no use of any chemical herbicides or pesticides such as glyphosate. In late October each year, a compound fertilizer (N: P: K = 21:8:16) was applied at a rate of 700 kg/ha.

### 2.3 Measurement of tea growth index and survey of vegetation coverage

#### 2.3.1 Vegetation cover measurement

This study utilized a 1 m × 1 m plot marked with a 10 cm scale on the edges. Five tea rows per treatment were selected, with three sample frames randomly placed in each row, and three replicates recorded. Spectral measurements and vertical photography were conducted in the study area during mid-April, August, and December of 2019 and 2020, between 10:00 a.m. and 2:00 p.m. under clear, cloudless conditions. The camera and spectrometer were set at the same height. Light measurement was performed using the handheld Green Seeker canopy spectrometer from NTech, United States, synchronized with vertical photography using a Pentax K5 DSLR camera, fixed at an 18 mm focal length and a 76° field of view, covering the entire treated area (Li et al., 2015).

#### 2.3.2 Measurement of tea tree growth indicators (2021–2022)

In mid-April of each year, the following parameters were measured according to the methodology of Zhang et al. (2023): tree height, canopy width, bud count, leaf area, chlorophyll SPAD value of the second functional leaf, internodal length between the second and third leaves, and fresh and dry weight of leaves. Specifically: Tree Height: Measured from the root collar (ground level) to the highest point of the crown where branches are most abundant. A total of three tea rows are measured, with five random replicate points selected in each row. Canopy Width: The diameter measured between the sides with the most lateral branches. Bud Count: Using a 1 m × 1 m frame placed over the harvesting surface to count the number of tea buds per unit area (visible buds only). Leaf Area: Thirty third functional leaves were randomly selected, their length and width measured, and the area calculated as Length × Width × 0.7. Chlorophyll Content (SPAD): Measured with a chlorophyll meter (TYS-N) on the second functional leaf of new shoots, with 10 replicates per plot. Internodal Length: The distance between the second and third functional leaves was randomly measured, with 10 replicates per plot. Fresh weight: Randomly select 100 standard samples of one bud with three leaves and weigh them. This process is repeated three times. Dry weight: These 100 samples are then dried in an oven at 105°C to a constant weight before weighing.

### 2.4 Soil sample collection

Soil samples were collected in mid-April using the five-point sampling method described by Zhong et al. (2019). Samples were taken from a depth of 10–15 cm at five points along each row, with three rows sampled and mixed evenly. Samples were preserved on ice, transported to the laboratory, sieved, and stored at −80°C and 4°C, respectively.

### 2.5 Determination of soil physicochemical properties and enzyme activities

The measurements of total nitrogen (TN), total phosphorus (TP), total potassium (TK), soil organic matter (SOM), pH, polyphenol oxidase (PPO), peroxidase (POD), acidic protease (ACPT), and cellulase (CE) were conducted according to the methods described by Liu J. et al. (2021).

### 2.6 Total DNA extraction from soil

Total soil DNA was extracted using the BioFast Soil Genomic DNA Extraction Kit (BioFlux, Hangzhou, China). The purity of DNA was verified by 1% agarose gel electrophoresis, and the concentration was measured using a NanoDrop2000C Spectrophotometer (Thermo Scientific, Massachusetts, United States). DNA of adequate quality was used for high-throughput sequencing analysis of the rhizosphere microbial community.

### 2.7 High-throughput sequencing analysis

PCR amplification of soil samples 16S rDNA (primers 341F and 806R) and ITS rDNA (primers 1737F and 2043R) was performed using an ABI GeneAmp^®^ 9700 PCR system (Supplementary Table S1). Subsequent sequencing was conducted using the Illumina Hiseq platform. Sequencing data were processed on the Qiime platform,^1^ where low-quality sequences with an average quality score below 20 (Q < 20) and shorter than 100 bp were removed to obtain effective tags. Clustering of all effective tags from the samples was done using Uparse, typically grouping sequences at 97% similarity into Operational Taxonomic Units (OTUs). Further species annotation of OTUs was performed using the Qiime’s blast method against the Unite database (v7.2) to determine microbial abundance at different taxonomic levels.

### 2.8 Data analysis

The soil physicochemical data are initially processed using Microsoft Office 2021, followed by significance analysis (ANOVA and LSD, *p* < 0.05) conducted with IBM SPSS software (version 26). Soil microbial community data are normalized and then analyzed for *α*-diversity analysis and *β*-diversity using the Bray-Curtis algorithm. Diversity indices are graphically represented using GraphPad Prism (version 7.0). Co-occurrence networks are computed using the “igraph” package in R software (version 4.3) with correlations determined by Spearman’s rank (∣r∣ > 0.7, *p* < 0.01), and visualized using Gephi (version 0.9.7) and Cytoscape (version 3.9.1). Functional predictions of bacterial and fungal communities in the soil are carried out based on the FAPROTAX and FUNGuild databases, respectively. Structural Equation Modeling (SEM) is performed using IBM SPSS Amos (version 28). Unless otherwise specified, other graphical analyses are conducted using R software (version 4.3).

## 3 Results and discussion

### 3.1 Effects of green manure cover on physical and chemical properties and enzyme activities of tea plant soil

**Figure 2:**
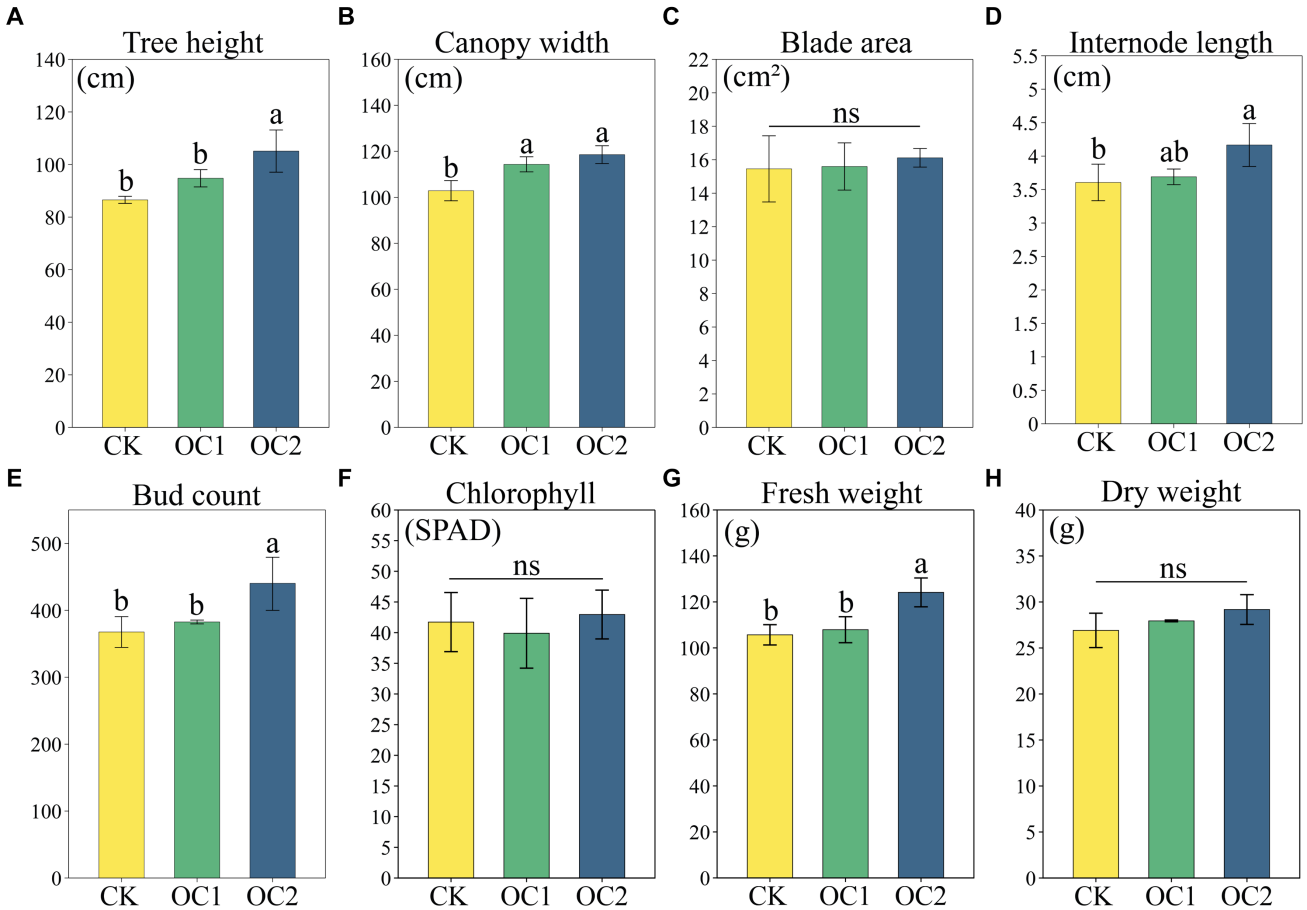
Tea plant growth and yield under different treatments. **(A)** Tree height. **(B)** Canopy width. **(C)** Blade area. **(D)** Internode length. **(E)** Bud count. **(F)** Chlorophyll. **(G)** Fresh weight. **(H)** Dry weight. Different letters denote significant differences (LSD, *p* < 0.05).

Green manure cover cropping, as a typical soil ecological positive feedback strategy, had a significant promoting effect on plant growth. The results showed that the advantages of green manure were significantly affected by the planting duration. Compared to the control (CK), the one-year cover (OC1) showed no significant differences in tea plant growth indicators except for canopy width. However, after two-years cover (OC2), the tree height, canopy width, internode length, bud count, and fresh weight of tea plants were all significantly increased (*p* < 0.05) (Figure 2). This indicates that green manure cover can significantly promote tea plant growth, which is consistent with previous research findings (Ma et al., 2021). Additionally, leaf area and chlorophyll values were less affected by green manure cover, with no significant changes observed. It is speculated that this is because the green manure model has a greater impact on the tea plant’s root system and above-ground branches (Chen et al., 2022), while leaf area (i.e., leaf size) is more influenced by the tea plant variety and nitrogenous fertilizer, leading to no significant differences (Karakaya and Dikilitas, 2018; Luo et al., 2023).

**Figure 3:**
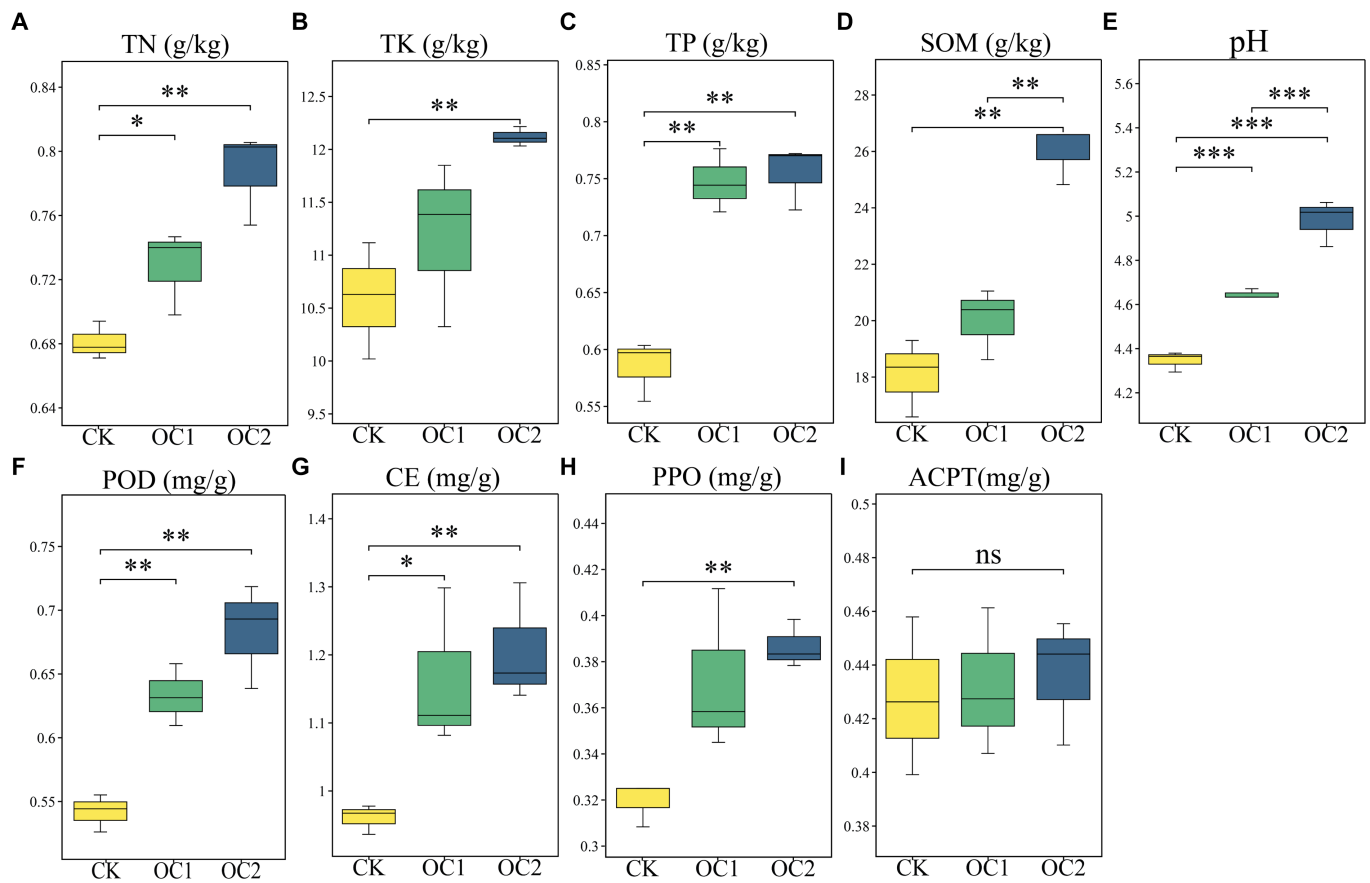
Changes in the physicochemical properties and enzyme activities of tea tree soil under different treatments. **(A)** Total nitrogen. **(B)** Total potassium. **(C)** Total phosphorus. **(D)** Soil organic matter. **(E)** pH. **(F)** Peroxidase. **(G)** Cellulase. **(H)** Polyphenol oxidase. **(I)** Acidic protease. Different numbers of asterisks indicate levels of statistical significance: * for *p* < 0.05, ** for *p* < 0.01, *** for *p* < 0.001.

The plant growth is closely related to soil nutrient status, with organic matter and pH serving as crucial indicators for evaluating soil fertility and nutrient status, closely linked to soil nutrient status (Yang et al., 2019). This study found that, compared to the control (CK), continuous coverage with green manure significantly increased soil total nitrogen, total phosphorus, total potassium, organic matter, and pH levels (*p* < 0.05). Organic matter can significantly enhance the soil’s water-holding capacity and moisture retention ability. Moreover, research has shown that the increase in organic matter can help alleviate soil acidification caused by continuous cropping of tea plants (Sokol et al., 2022). Similarly, the activities of cellulase, peroxidase, and polyphenol oxidase were significantly enhanced, whereas acid proteinase activity showed no significant change (Figure 3). These findings align with previous research (Adekiya, 2019). The significant increase in these soil enzymes mainly originates from soil microbes and root exudates, which are responsible for the degradation of animal and plant residues, cellulose, and polyphenols (Hu et al., 2022). These changes suggest that soil in mountain tea plantations covered with *O. japonicus* has improved capabilities for degrading large organic molecules and an enhanced nutrient status, which progressively increase with the duration of cover.

### 3.2 Green manure coverage changed the diversity and structure of soil microbial community

**Figure 4:**
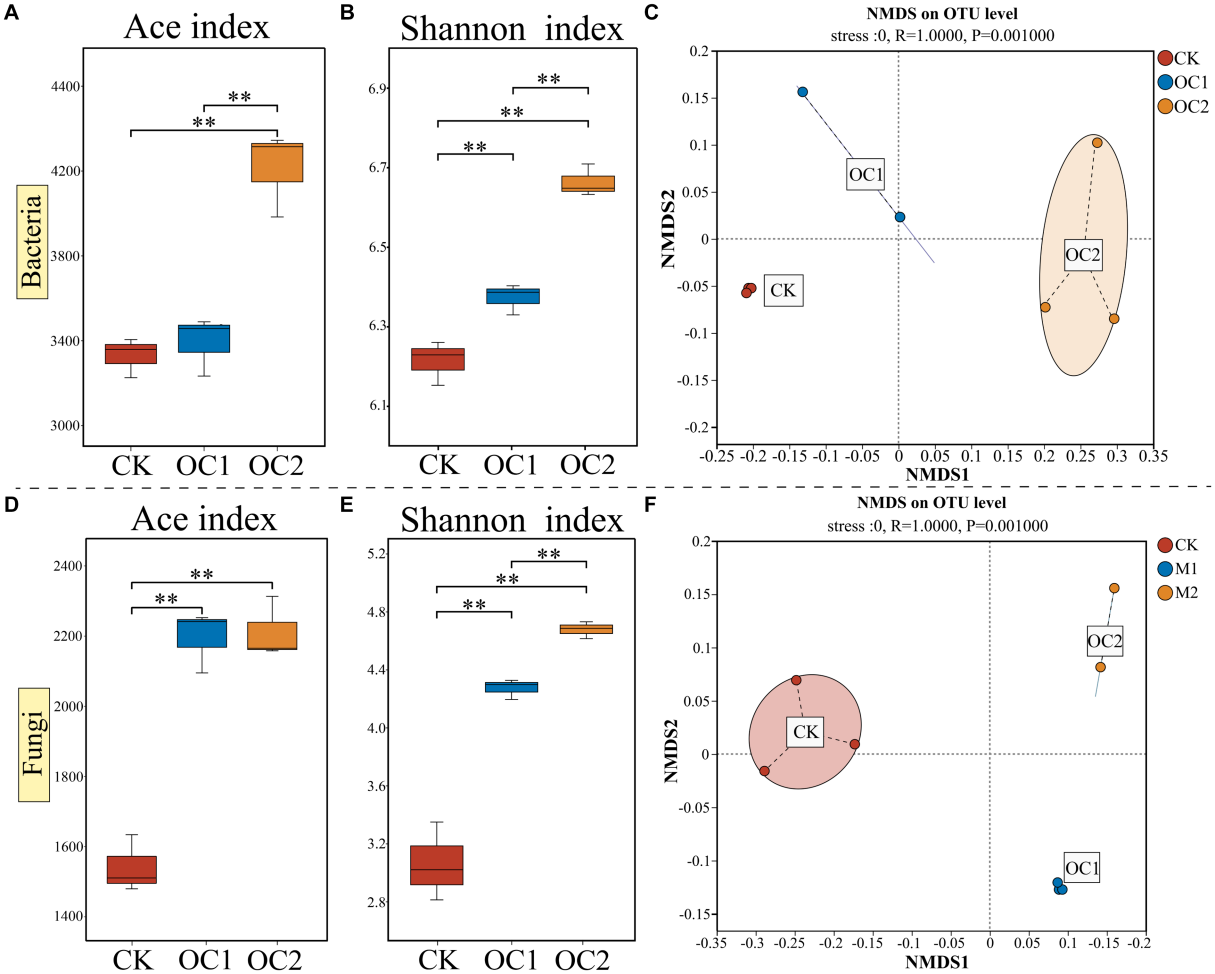
Changes in alpha diversity (Ace and Shannon) and beta diversity (NMDS) of soil microbial communities under different treatments. **(A)** Bacterial ace index. **(B)** Bacterial Shannon index. **(C)** Bacterial NMDS analysis. **(D)** Fungal ace index. **(E)** Fungal Shannon index. **(F)** Fungal NMDS analysis. Different numbers of asterisks indicate levels of statistical significance: * for *p* < 0.05, ** for *p* < 0.01, *** for *p* < 0.001.

Soil microorganisms are a crucial component of the soil ecosystem, as they convert insoluble organic matter into soluble substances, playing a key role in the transformation and cycling of soil organic matter (Wang et al., 2021). In this study, high-throughput sequencing of soil microorganisms was conducted. The results indicated that the diversity of bacteria and fungi (measured by the ACE index and Shannon index) in the OC2 treatment was significantly higher than in OC1 and CK (*p* < 0.01), with microbial diversity increasing significantly with the duration of cover (Figure 4). NMDS analysis showed clear differences between the microbial communities (both bacterial and fungal) in soils covered with green manure and those in conventional tea plantations, with bacterial community differences expanding over the years of cover. Previous research has demonstrated that the input of plant litter can increase soil microbial diversity (Wardle et al., 2006; Chapman et al., 2013; Delgado-Baquerizo et al., 2016). Moreover, some studies have indicated that microbial functional diversity can enhance soil ecosystem functions, such as disease suppression (Mallon et al., 2015; Poveda et al., 2021) and decomposition and nutrient cycling (Gielnik et al., 2021; Mącik et al., 2022). A diversified community can decompose residues more quickly and utilize nutrients more efficiently (Vidal et al., 2021). Therefore, these findings suggest that the use of *O. japonicus* as a cover crop in tea plantations progressively builds a unique microbial community, which can have profound implications for soil health and productivity.

**Figure 5:**
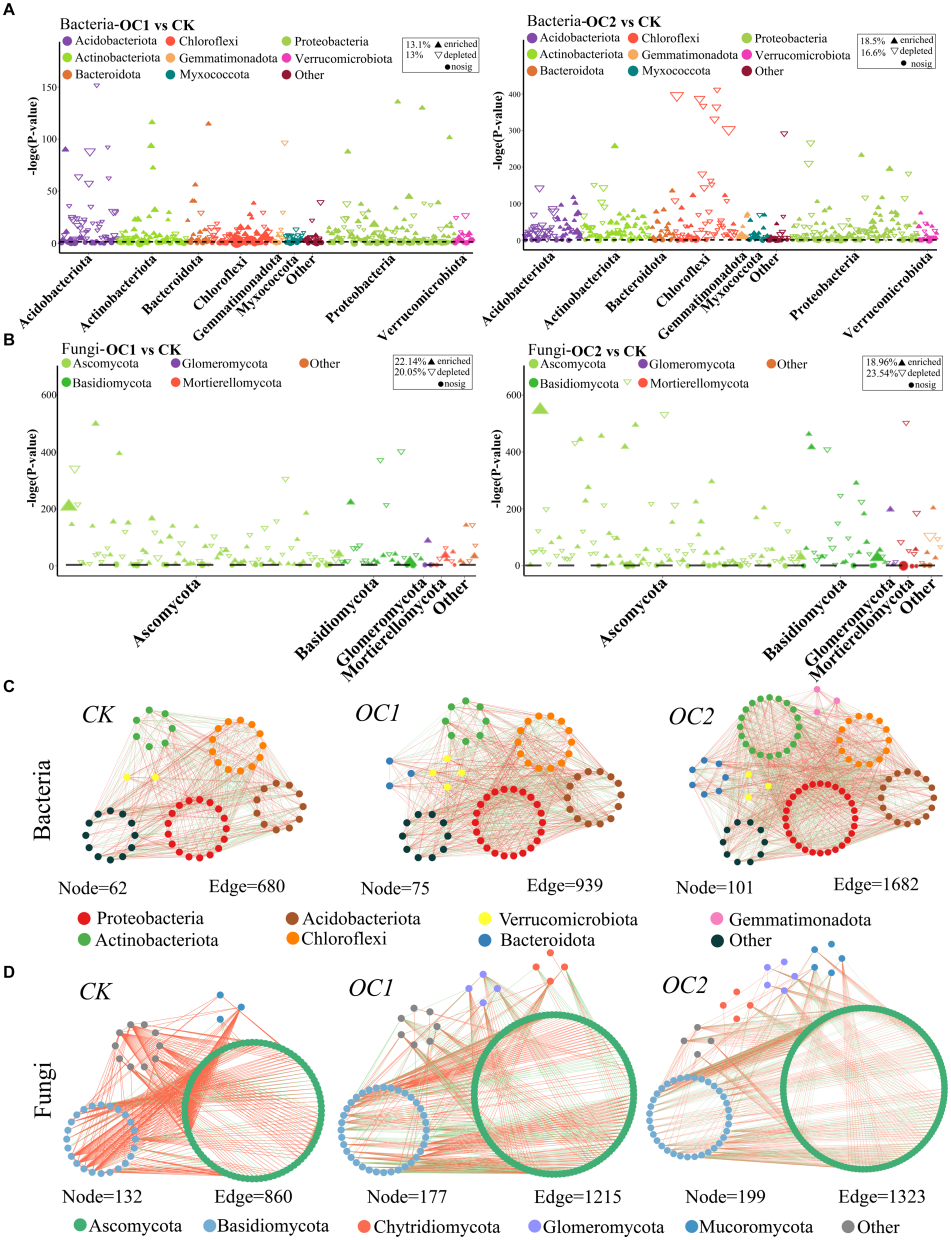
Co-occurrence networks and Manhattan plots of species in soil microbial communities under different treatments. The co-occurrence networks of soil bacteria **(A)** and fungi **(B)** were constructed based on the Spearman correlation algorithm among microbial communities. The connections between nodes indicate significant correlations (∣r∣ > 0.7, *p* < 0.1), and different colors represent different phyla. The Manhattan plots show the enrichment or downregulation of OTUs in the microbial communities of soil bacteria **(C)** and fungi **(D)** under different treatments. Each solid upward triangle represents an enriched OTU (*p* < 0.05), each hollow downward triangle represents a downregulated OTU, and each circle represents an OTU with no significant change.

Manhattan analysis reveals that continuous coverage with *O. japonicus* significantly increased the species richness of Proteobacteria and Actinobacteria in the soil (Figures 5A,B). Compared to the control (CK), the proportion of significantly upregulated OTUs in OC1 and OC2 for Proteobacteria were 40.25 and 48.1% of their total OTUs, respectively; for Actinobacteria, these figures were 33.7 and 61.4%. In terms of fungi, Ascomycota and Basidiomycota were the main phyla affected by the cover treatments, with significantly upregulated OTUs accounting for 45.6 and 40.95% of their total OTUs in OC1 and OC2 for Ascomycota, respectively, and 48.3 and 48.38% for Basidiomycota (Supplementary Table S2). Proteobacteria and Actinobacteria contain many copiotrophic genera, which are prevalent in nutrient-rich soils and play critical roles in cellulose degradation and humus formation (Ventura et al., 2007; Delmont et al., 2018). Ascomycota and Basidiomycota are also key decomposers of soil organic matter, lignin, and cellulose (de Oliveira Rodrigues et al., 2020; Zhan et al., 2021).

Notably, co-occurrence network analysis indicated that with increasing years of green manure coverage, The number of co-occurrence network nodes in Proteobacteria, Bacteroidetes, Ascomycota, and Basidiomycota all increased independently, representing a rise in microbial diversity and symbiotic levels (Figures 5C,D). Furthermore, the modularity values of the co-occurrence networks increased with the duration of green manure coverage (for bacteria, values progressed from 0.57 < 0.607 < 0.651; for fungi, from 0.18 < 0.24 < 0.28). Modularity refers to the ecological niches within microbial communities (Faust, 2021). In this study, the coverage with green manure enhanced the interactions among key microbial groups in the soil, with higher modularity values potentially associated with strengthened ecological symbiotic relationships under continuous green manure coverage. This implies a progressively more structured and cooperative microbial ecosystem, which may further enhance soil health and productivity.

### 3.3 Effects of green manure cover on soil microbial community function and key microflorae

**Figure 6:**
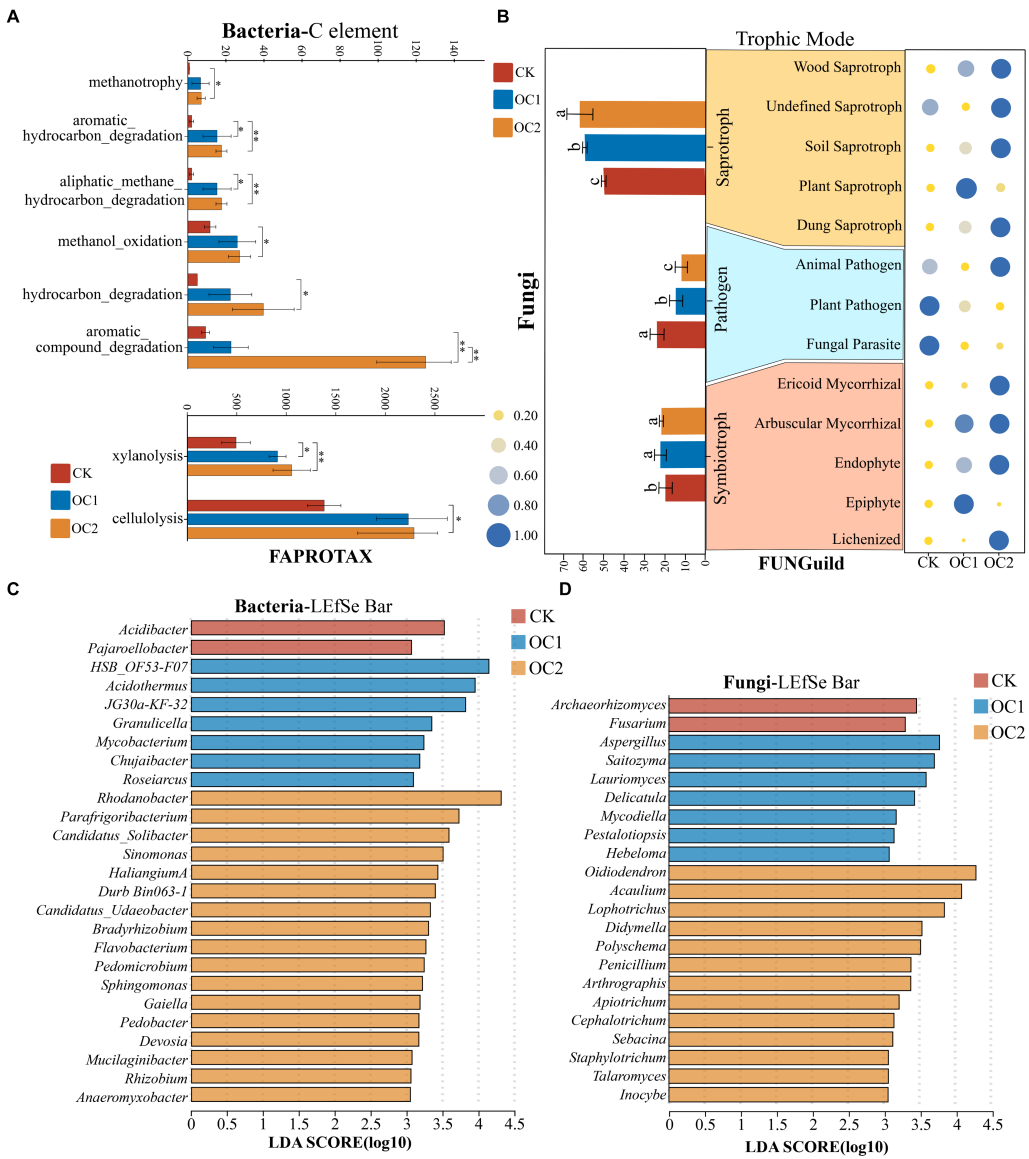
Functional prediction and LEfSe analysis of soil microbial communities under different treatments. **(A)** Functional analysis of the soil bacterial community based on the FAPROTAX database. Different numbers of asterisks indicate levels of statistical significance: * for *p* < 0.05, ** for *p* < 0.01, *** for *p* < 0.001. **(B)** Functional analysis of the soil fungal community based on the FUNGuild database. **(C)** Key bacterial genera in soils under different treatments identified through LEfSe analysis (LDA > 3). **(D)** Key fungal genera in soils under different treatments identified through LEfSe analysis (LDA > 3).

The alteration in microbial community structure has consequential implications for its ecological functions. In this study, functional annotation of soil bacteria across different treatments was performed using the FAPROTAX database. In total, 1,242 OTUs (out of a total of 5,725 OTUs, with an annotation rate of 21.69%) were annotated to 49 functions. Significant changes in bacterial functions were observed under different treatments (*p* < 0.05), indicating that the predictions for all OTUs are statistically significant. The analysis revealed that functions related to substance degradation in bacteria changed significantly under the cover treatments (Figure 6A). The OC2 treatment significantly increased the proportions of functions such as aromatic hydrocarbons degradation, aliphatic methane hydrocarbon degradation, methanol oxidation, hydrocarbon degradation, aromatic compound degradation, xylanolysis, and cellulolysis. The nutritional modes of fungi predominantly encompassed symbiotrophs, saprotrophs, and pathogens. Under the cover of green manure, the abundance of symbiotrophs and saprotrophs significantly increased, while the proportion of pathogenic fungi continually decreased (Figure 6B). Further analysis of the main fungal nutritional modes revealed that with increasing cover duration, the abundance of Wood Saprotroph, Soil Saprotroph, Dung Saprotroph, Ericoid Mycorrhizal, and Arbuscular Mycorrhizal in the soil fungal community continuously increased, while the abundance of Plant Pathogen and Fungal Parasite continuously decreased.

According to Van Elsas et al. (2012), higher microbial diversity can enhance the soil’s ecological resilience against pathogen invasion. This enhancement is likely due to the more diverse metabolic potential within the soil microbial community, which intensifies internal competition for environmental resources, thereby reducing the ecological niches available for pathogen invasion (Mallon et al., 2018). More diverse microbial activity also helps form and stabilize soil aggregates, promoting the cementation of soil mineral particles and creating a more stable aggregate structure. Additionally, the cover of green manure crops protects the soil surface, reducing the erosive effects of rainfall. The presence of cover reduces the speed and erosive force of water flow, protecting soil structure and facilitating the restoration of soil fertility. This suggests that the introduction of green manure such as *O. japonicus* not only improves soil health through enhanced nutrient cycling and organic matter decomposition but also potentially creates a more competitive environment that is less hospitable to pathogenic invasions, thereby contributing to the overall resilience and stability of the soil ecosystem.

LEfSe (Linear discriminant analysis Effect Size) analysis indicated that there are significant differences in 26 bacterial genera and 22 fungal genera (LDA > 3; Figures 6C,D). Bacterial genera such as *Rhodanobacter*, *Sinomonas*, *Haliangium*, *Bradyrhizobium*, *Sphingomonas*, *Gaiella*, and *Rhizobium* had notably higher abundances in OC2 compared to OC1 and CK. Previous studies have demonstrated that genera like *Sphingomonas*, *Candidatus Solibacter*, *Sinomonas*, *Haliangium*, and *Gaiella* have the capability to degrade organic matter in soil, thereby facilitating nutrient cycling (Kim et al., 2015; Sun et al., 2016; Asaf et al., 2020; Bai et al., 2020; Zhang et al., 2021). *Bradyrhizobium* and *Rhizobium* are well-known plant growth-promoting bacteria that play significant roles in soil ecological stability (Barbosa et al., 2021; Shang and Liu, 2021; Yang et al., 2022). Furthermore, continuous coverage with green manure significantly increased the abundance of fungal genera such as *Oidiodendron*, *Penicillium*, *Apiotrichum*, and *Talaromyces*. These fungi are also highly efficient at degrading organic matter (Sun et al., 2020; Méndez-Líter et al., 2021; Yan-Hua and Li-Fu, 2021). Previous research has demonstrated that green manure cover can increase nutrient content in soil. The underlying ecological process might be attributed to the green manure enhancing the abundance of beneficial soil microbes. The activity of key microbial-derived enzymes such as cellulases and peroxidases activates readily available nutrients for plant uptake. This process further releases a large number of polyphenolic polymers and alkaloids contained in the green manure residues, thereby enhancing the activity of polyphenol oxidases in the soil (Guo et al., 2021), which improves the soil environment.

Additionally, previous studies have shown that under monoculture conditions, the decomposition of tea leaf litter leads to the accumulation of allelochemicals such as polyphenols in the soil, which are major contributors to the issue of replant disease in tea plants (Arafat et al., 2017). It can be inferred that under green manure cover, the enhanced degradation capabilities of the soil microbial community for organic matter and aromatic compounds are beneficial for improving the soil nutritional environment and promoting tea plant growth.

### 3.4 Correlation analysis between microbial communities and soil properties

**Figure 7:**
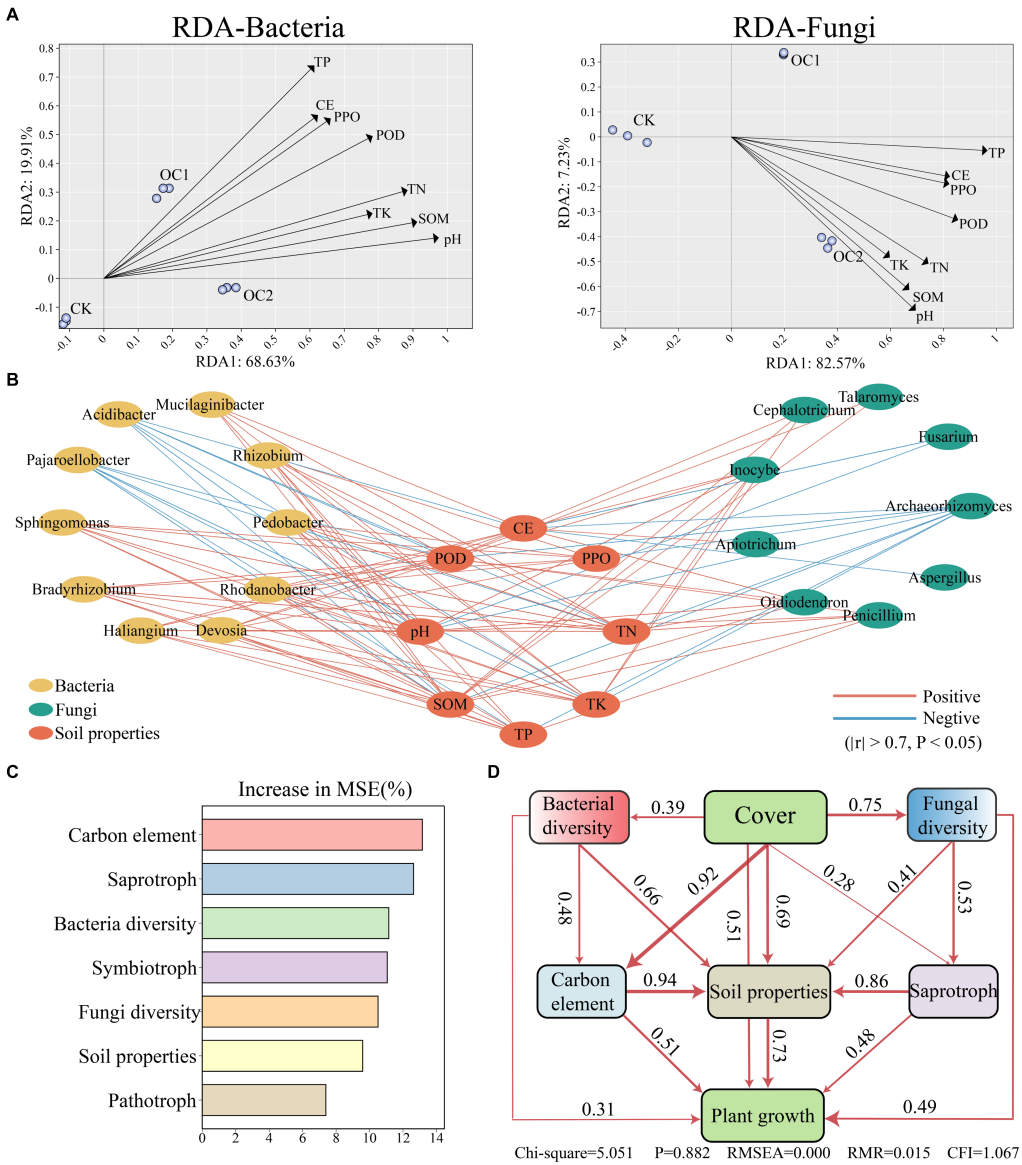
Interaction analysis of soil properties, soil microbial communities, and tea tree growth conditions. **(A)** Interaction analysis between soil microbial communities and soil properties based on the RDA (Redundancy Analysis) algorithm. **(B)** Correlation analysis between soil properties and significantly different microbial genera calculated using the Spearman algorithm (∣r∣ > 0.7, *p* < 0.05). **(C)** Analysis of the weight differences in various microbial functions under green manure cover using a random forest regression model. “Increase in MSE (%)” represents the weight value, with higher values indicating greater importance of the variable. **(D)** Analysis of the overall interaction effects between different variables using a structural equation model.

A redundancy analysis (RDA) integrating soil microbial communities and soil properties showed that the first two RDA axes (RDA1 and RDA2) explained 88.54% of the variation in soil bacterial communities (Figure 7A) and 89.8% of the variation in soil fungal communities (Figure 7B). RDA illustrates the correlation between soil properties and the microbial community under the cover of green manure. Apart from ACPT, other soil properties show a significant positive correlation with changes in the microbial community, and this effect intensifies with increased years of cover (Figure 7B). Further Spearman correlation analysis (Figure 7C) shows that beneficial microbes such as *Haliangium*, *Sphingomonas*, *Gaiella*, *Oidiodendron*, *Penicillium*, *Apiotrichum*, and *Talaromyce* have significant positive correlations with soil organic matter and pH.

Based on a random forest regression model, the primary factors influencing soil environment under the cover of green manure were identified as the carbon element, saprotrophs and microbial diversity (Figure 7D). Further analysis using a structural equation model (SEM) revealed that green manure cover has a direct positive impact on soil properties (*λ* = 0.69) and plant growth (λ = 0.51). It also positively affects bacterial diversity (λ = 0.39), fungal diversity (λ = 0.75), saprotrophs (λ = 0.28), and the carbon element (λ = 0.92). Additionally, changes in the carbon element (λ = 0.94) have the most direct impact on soil organic matter, indicating a higher sensitivity of organic matter to the carbon cycle compared to other factors. This highlights the integral role of carbon dynamics and saprotrophic activities in enhancing soil quality and promoting plant growth under green manure cover.

Previous research has shown that the use of green manure intensifies interactions between soil properties and the biotic community, with organic matter and pH being the dominant factors in these interactions (Sun et al., 2015; Das et al., 2017). Most importantly, an increase in organic matter significantly mitigates soil acidification (Jayalath et al., 2016). This study indicates that the introduction of green manure results in a more diverse input of carbon sources, leading to the enrichment of various degradative microbial genera such as *Haliangium*, *Sphingomonas*, *Penicillium*, *Oidiodendron*, and *Talaromyces*. This enrichment accelerates the transformation of plant-derived carbon sources and nutrients in soil organic matter. This process provides more substrates for soil enzyme activity, thereby enhancing the chelation of base cations in the soil (Xu et al., 2017), reducing the loss of base cations, alleviating soil acidification, and ultimately promotes the growth of tea plants. Moreover, the enrichment of beneficial bacteria under green manure cover reduces ecological niches, which can decrease the abundance of host-specific pathogens, thereby lowering the risk of soil-borne diseases. Overall, the use of *O. japonicus* as green manure cover enriches beneficial microbial communities and promotes the carbon cycle, thus significantly improving the soil environment. Therefore, when developing new strategies to improve tea tree health by promoting the abundance of beneficial microbes to suppress pathogen activity, the findings mentioned above should be considered.

## 4 Conclusion

With the increasing duration of *Ophiopogon japonicus* cover, the diversity of soil microbial communities and the complexity of co-occurrence networks in mountainous tea plantation significantly increased. Functional microbial groups related to the carbon cycle were significantly enriched, enhancing the soil’s ability to degrade organic matter such as cellulose and aromatic compounds. This accelerated the decomposition of organic matter and the activation and release of readily available nutrients, raising the soil pH and significantly promoting tea plant growth. Therefore, in the practice of tea cultivation, the use of *Ophiopogon japonicus* as a cover crop for three-dimensional mountain tea plantation cultivation offers a way to promote the recycling of energy and materials at multiple levels. This approach can provide new ideas for improving the ecological environment of mountain tea plantations.

## Data Availability

The raw data supporting the conclusions of this article will be made available by the authors, without undue reservation.

## Footnotes

^1^ http://qiime.org/scripts/assign_taxonomy.html
