# Supplementary material for: Green manure (Ophiopogon japonicus) cover promotes tea plant growth by regulating soil carbon cycling
Source: Front Microbiol. 2024 Sep 18;15:1439267. doi: 10.3389/fmicb.2024.1439267 (PMC11447704; doi:10.3389/fmicb.2024.1439267)
Supplement: Supplementary file 1 [file Data_Sheet_1.docx]

| Target | Primers | Primer sequence (5’- 3’) | PCR reaction condition |
| --- | --- | --- | --- |
| 16S | 338F  806R | ACTCCTACGGGAGGCAGCAG  GGACTACHVGGGTWTCTAAT | 95°C for 3 minutes, 27 cycles of (30 s at 95°C, 30 s at 55°C and 45 s at 72°C, 72°C for 10 minutes) |
| ITS | ITS2-2043R  ITS5-1737F | GCTGCGTTCTTCATCGATGC  GGAAGTAAAAGTCGTAACAAGG | 95°C for 5 minutes, 35 cycles of (30 s at 94°C, 30 s at 52°C, for 30 s at 72°C，72°C for 10 minutes), |

**Table S1** Primers and PCR thermal cycling procedures

**Table S2** The number of OTU changes at the phylum level.

| **OC1 VS CK** （Bacteria Phylum） | **Significant change** | **Up** | **Lower** |
| --- | --- | --- | --- |
| Proteobacteria | 79 | 53 | 26 |
| Actinobacteriota | 37 | 25 | 12 |
| Chloroflexi | 38 | 17 | 21 |
| Acidobacteriota | 43 | 7 | 36 |
| Bacteroidota | 15 | 11 | 4 |
| Verrucomicrobiota | 11 | 6 | 5 |
| Myxococcota | 14 | 2 | 12 |
| Other | 10 | 3 | 7 |
| Gemmatimonadota | 6 | 1 | 5 |
| Patescibacteria | 3 | 3 | 0 |
| Desulfobacterota | 2 | 1 | 1 |
| Methylomirabilota | 1 | 0 | 1 |
| **OC2 VS CK**（Bacteria Phylum） | **Significant change** | **Up** | **Lower** |
| Proteobacteria | 111 | 65 | 46 |
| Actinobacteriota | 68 | 45 | 23 |
| Chloroflexi | 57 | 12 | 45 |
| Acidobacteriota | 55 | 24 | 31 |
| Bacteroidota | 20 | 18 | 2 |
| Verrucomicrobiota | 14 | 8 | 6 |
| Myxococcota | 18 | 10 | 8 |
| Other | 12 | 2 | 10 |
| Gemmatimonadota | 11 | 9 | 2 |
| Patescibacteria | 5 | 1 | 4 |
| Desulfobacterota | 2 | 2 | 0 |
| Methylomirabilota | 1 | 1 | 0 |
| **OC1 VS CK** （Fungi Phylum） | **Significant change** | **Up** | **Lower** |
| Ascomycota | 92 | 47 | 45 |
| Basidiomycota | 26 | 11 | 15 |
| Mortierellomycota | 9 | 6 | 3 |
| Rozellomycota | 3 | 0 | 3 |
| Glomeromycota | 3 | 1 | 0 |
| Mucoromycota | 2 | 0 | 2 |
| Chytridiomycota | 2 | 1 | 1 |
| Olpidiomycota | 1 | 0 | 1 |
| **OC2 VS CK**（Fungi Phylum） | **Significant change** | **Up** | **Lower** |
| Ascomycota | 105 | 45 | 59 |
| Basidiomycota | 31 | 14 | 17 |
| Mortierellomycota | 9 | 2 | 7 |
| Glomeromycota | 4 | 2 | 2 |
| Mucoromycota | 4 | 2 | 2 |
| Rozellomycota | 3 | 1 | 2 |
| Olpidiomycota | 1 | 1 | 0 |
| Chytridiomycota | 1 | 1 | 0 |


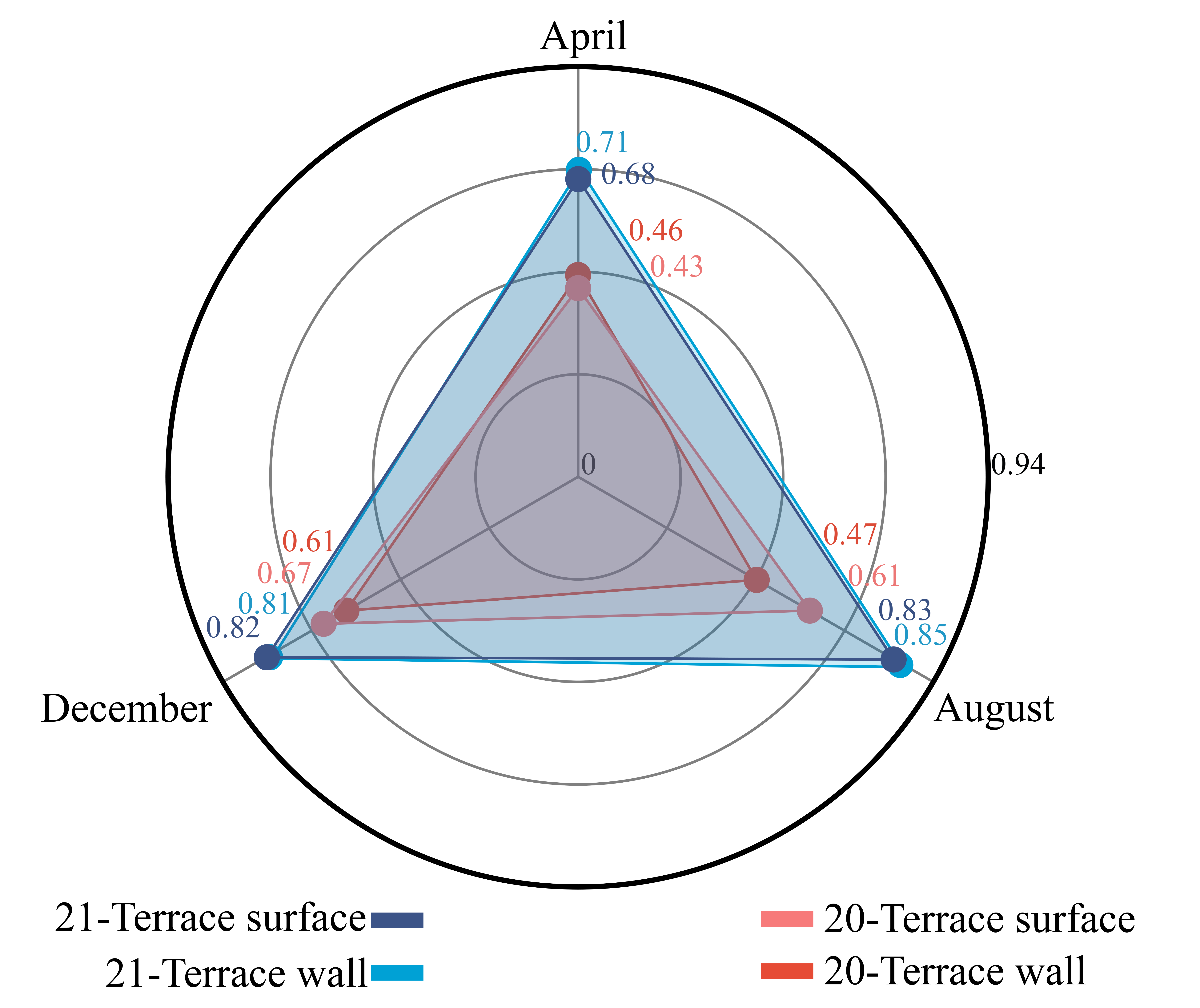


**Figure S1**. Vegetation coverage rate of tea garden in 2020-2021 (%)
